# Success in Life

**Published:** 1863-02

**Authors:** 


### SUCCESS IN LIFE.

If to obtain wealth is success, we see men around us who have accumulated fortunes, who have no remarkable talent, no special high moral character; in fact, in general intelligence, in elevation of sentiment, in breadth of view, they are pitifully deficient; while men, immeasurably their superiors in every great and good quality, have never made and saved a dollar.

Then again, every now and then, we meet with a man who seems

